# Floppy iris. How is floppy iris syndrome managed? What do urologists and ophthalmologists say?

**DOI:** 10.22336/rjo.2024.54

**Published:** 2024

**Authors:** Bogdan Bumbuluț, Dan Mircea Stănilă

**Affiliations:** 1 Sibiu County Emergency Clinical Hospital, Sibiu, Romania,; 2 Dr. Stănilă Medical Centre, Ofta Total Clinic, “Lucian Blaga” University of Sibiu, Romania

**Keywords:** Floppy iris, benign prostatic hypertrophy, tamsulosin, IFIS = Intraoperative Floppy Iris Syndrome, BPH = Benign Prostatic Hyperplasia, NSAIDs = Non-Steroidal Anti-Inflammatory Drugs

## Abstract

**Introduction:**

This study focuses on the effect of chronic treatment on the eye, regarding the so-called Intraoperative Floppy Iris Syndrome (IFIS) or Floppy Iris Syndrome, which can occur during cataract surgery.

**Aim:**

The study aimed to establish the influence of tamsulosin, used in benign prostatic hyperplasia (BPH) treatment on the iris, during cataract surgery, considering the increased incidence of both conditions in older age.

**Methods:**

This study included one hundred male patients, operated on for cataracts at the Ofta Total Clinic and Dr. Stănilă Medical Centre, in Sibiu, out of 601 patients operated on for cataracts between February and October 2022. Of the 100 patients, 24 used medication for BPH. 5 patients used prostamol, a phytotherapeutic preparation, which is an extract from Serenoarepens, and the remaining 19 used tamsulosin, which is an alpha-blocker, most commonly used in the treatment of BPH, considered the first-line treatment option.

**Results:**

In 15 patients, we intraoperatively managed, a medium mydriasis through pharmacological dilation, including intracameral administration of phenocaine and mechanical dilation or stripping. In 4 patients it was necessary to apply iris dilators. Due to the small pupil in 2 patients, we caught the iris in the phacoemulsification probe, and a small, incomplete iris coloboma was formed. Sometimes, there was a turnover of Descemet in 4 patients. The pupil remained semi-dilated and slightly areflective in the patients to whom we applied iris hooks. The patients’ visual acuity was satisfactory, between 0.9 and 0.6.

**Discussions:**

The topic gives rise to many discussions. It seems that stopping the administration of tamsulosin for a short time does not help the occurrence of IFIS, because the iris lesions seem irreversible. Patients at risk of developing cataracts should be evaluated and possibly referred to an ophthalmologist to determine surgery before starting treatment for BPH and to competently assess the administration of this medication.

**Conclusions:**

Collaboration between urologists and ophthalmologists is required for patients prone to the appearance of cataracts since both conditions are frequently encountered in elderly patients.

## Introduction

Chronic treatment of patients with benign prostate hypertrophy (BPH) with tamsulosin can lead to changes in the eyeball with unfavorable repercussions on the various structures of the eyeball and especially on the iris during cataract surgery. It was discovered in 2005 and is associated with the use of treatment with alpha-blockers under the name Iris Flaccid Intraoperative Syndrome (IFIS) [1]. IFIS can be mild, moderate, or severe and differs from patient to patient.

### The influence of tamsulosin on the iris

This study focuses on the effect on the eye, the so-called Intraoperative Floppy Iris Syndrome (IFIS) or Floppy Iris Syndrome, which can occur during cataract surgery [2].

On the other hand, one of the most used drugs in BPH is tamsulosin (Fokusin, Tamsulosin, Aurobindo, Tanis, or Tamsol). But what do the urologists say? This drug is part of the class of alpha-blockers also known as antagonists of alpha-adrenergic receptors. The beneficial effect on the prostate is the relaxation of the muscle fibers, the bladder muscles, and the facilitation of urination [2]. What do ophthalmologists say? Tamsulosin produces miosis on the iris, which becomes flaccid and tends to prolapse. This side effect bothers ophthalmologists during cataract surgery, sometimes with serious complications [2].

The study aimed to establish the influence of tamsulosin, used in BPH treatment, on the iris, during cataract surgery, considering the increased incidence of both diseases in older age. What are the advantages of the administration? Is there a balance between the urological advantages and the ophthalmological disadvantages?

## Materials and methods

**Fig. 1:**
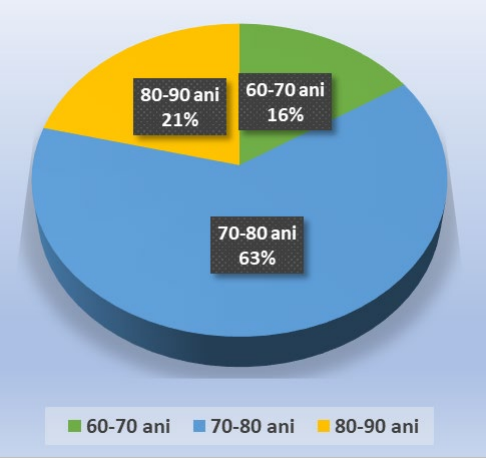
Distribution of patients according to age

Of 601 patients operated on for cataracts in 2022 (23.02.2022-22.10.2022), one hundred male patients were operated on for cataracts at Ofta Total Clinic, and Dr. Stănilă Medical Centre, in Sibiu. The enrolled patients’ age was between 60 and 89 years. The distribution by age groups showed that most patients were between 70-80 years old, 63%, followed by those between 80-90 and 60-70 years old (**Fig. 1**).

Out of the 100 patients, 24 used medicines for BPH. 5 patients used prostamol, a phytotherapeutic preparation, which is an extract from Serenoa repens, and the remaining 19 used tamsulosin, which is an alpha-blocker, most commonly used in the treatment of BPH, and considered the first-line drug. All patients operated on for cataracts were previously treated within the urology ward for urination disorders due to benign prostate hypertrophy. We followed the changes that occurred preoperatively, intraoperatively, and early postoperatively. All patients were operated on by the same surgeon with topical anesthesia by the phacoemulsification method with artificial lens implantation in the capsular bag.

## Results

This study focuses on the effect on the eye, the so-called Intraoperative Floppy Iris Syndrome (IFIS) or Floppy Iris Syndrome, which can occur during cataract surgery. In all patients, we had problems related to pupil dilation before surgery. In 15 patients, we managed a medium mydriasis intraoperatively by pharmacological dilation, including the intracameral administration of mydriatics and mechanical dilation or striping.

**Fig. 2:**
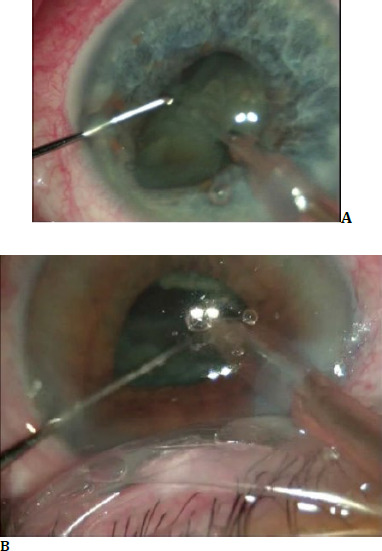
Iris responds to intraocular fluids

**Fig. 3:**
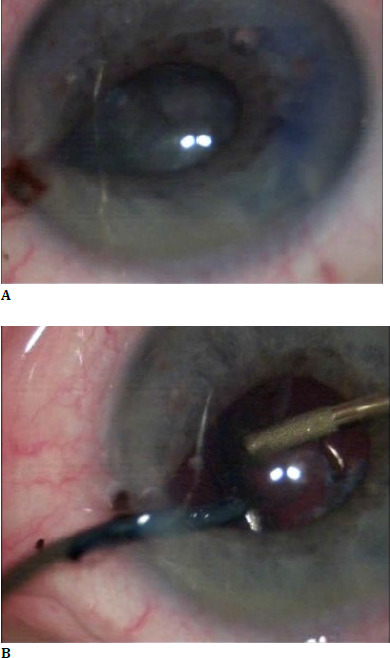
Iris prolapse through wounds

The floppy iris responds to intraocular fluids **(Fig.2 A, B**). It tends to iris prolapse through wounds **(Fig. 3 A, B**), and to the progressive contraction of the pupil.

**Fig. 4:**
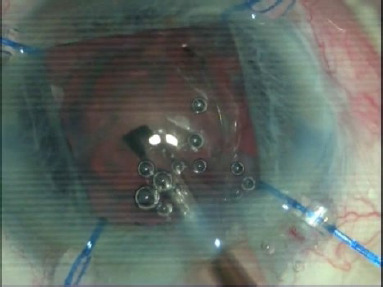
Iris dilators

Iris dilators needed to be applied in 4 patients **(Fig.4**).

**Fig. 5:**
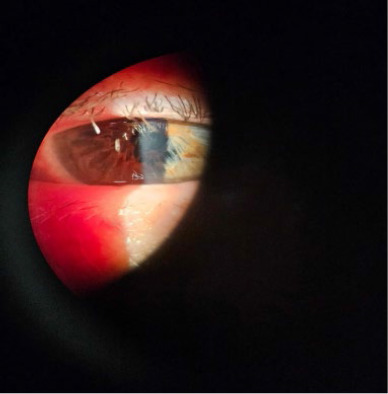
Incomplete iris coloboma

Due to the small pupil, in 2 patients we caught the iris in the phacoemulsification probe, and a small, incomplete iris coloboma was formed (**Fig. 5**). Sometimes, there was a turnover of Descemet in 4 patients. The pupil remained semi-dilated and slightly areflective in the patients to whom we applied iris hooks. The patients’ visual acuity was satisfactory, between 0.9 and 0.6. Due to the trauma of the iris in 2 patients, a uveal reaction occurred, easily controlled by treatment with steroidal anti-inflammatory drugs. We had no major complications such as retinal detachment or endophthalmitis [3].

## Discussions

The topic gives rise to many discussions. It seems that stopping the administration of tamsulosin for a short time does not help the occurrence of IFIS, because iris lesions seem irreversible [1].

Tamsulosin, together with other alpha-blockers - doxazosin, alfuzosin, terazosin, and silodosin - relaxes the smooth muscles of the prostate and bladder neck, decreasing the frequency of urination and improving the quality of life of urological patients. According to urologists, the floppy iris syndrome can also occur in females when administered in urinary disturbances or renal colic [3].

There is evidence that blocking α1 adrenergic receptors causes relaxation of the dilator muscle of the iris, with weak pupillary response. Furthermore, it has been suggested that long-term intake of α1-ARA may cause anatomical changes that are permanent and cannot be completely reversed with discontinuation of the medication and are maintained despite the use of preoperative pharmacological dilation with topical cyclopentolate/phenylephrine and ketorolac [1]. One study also reported drug-melanin interaction causing iris dilator muscle atrophy and, therefore, IFIS [4].

On the other hand, according to ophthalmologists, IFIS dramatically disrupts cataract surgery.

- A floppy iris ripples and moves excessively when fluids are injected into the eye during cataract surgery, leading to complications and stress for the surgeon.
- The pupil gradually becomes smaller as the procedure progresses. Even if the pupil is dilated before the cataract operation, it becomes miotic during the operation, and a pupil that is too small makes it particularly difficult to remove the cataract. The iris tends to come out through the incisions made at the beginning of the surgical intervention [4].
- Difficulties regarding pupil dilation occur in many patients. The edge of the pupil may show elasticity, and the entire iris will have a poor consistency regarding its structure due to poor muscle tone. IFIS can also cause damage to other tissues in the eye, including the retina, and lens capsule.

Advising patients to stop the administration of the drug before surgery may be of questionable value, according to the information presented in the literature. Dr. Chang noted that, in a large prospective multicentered study, stopping tamsulosin before surgery did not decrease the rate or severity of IFIS. Among survey respondents, 64% say they never stop tamsulosin preoperatively, compared to only 11% who routinely do so [4].

For patients with BPH and tamsulosin treatment, who are candidates for cataract surgery, it is important to discuss the side effects of alpha-blockers on the eye. Ophthalmological surgeons should inform patients taking tamsulosin before surgery that cataract surgery may not proceed normally and may give rise to complications, which are sometimes major [5].

### Complications

Several complications that may occur during cataract surgery such as photophobia (sensitivity to light) and glare, a deformed pupil, and weak pupillary dilation may be experienced if a person is at risk of IFIS. They can lead to an iris reaction with chronic uveitis phenomena, increased risk of uveitis, and retinal detachment.

Some problems can even endanger patients’ vision.

Can IFIS be prevented? Simply stopping the use of tamsulosin or other drugs that can cause IFIS before cataract surgery often does not prevent IFIS. Conversely, there may be greater risks for urological disease caused by discontinuation of the drug [6].

A recent study proposed that atropine sulfate 1% eye drops at 40 and 20 minutes before surgery significantly reduce the incidence of IFIS, especially its mild forms [4].

It is worth noting that many surgeons favor the use of preoperative topical non-steroidal anti-inflammatory drugs (NSAIDs) [7].

Eye surgeons can take precautions before and during surgery to reduce the risk of IFIS.

During surgery, the pupils must remain dilated to prevent IFIS. There are several techniques for keeping the pupil dilated. These may include approaches such as manual dilation with a special instrument and the use of certain medications.

Many surgeons use instruments called iris retractors, iris hooks, or expansion rings. They are installed through incisions before the operation and removed at the end of the procedure. Each instrument is designed to dilate the iris and keep the pupil dilated.

Experts consider the iris hook/retractor method the most reliable in IFIS prevention [8].

Other methods of controlling or preventing IFIS may include: administering medications such as preoperative atropine, intraoperative phenylephrine, or an epinephrine injection under the patient’s iris.

The viscoelastic fluids are designed to protect a layer of cells on the cornea, called the corneal endothelium. They also help maintain the anterior chamber. These substances are usually injected during surgery, but additional doses may be needed to be effective in IFIS.

While these options can help prevent IFIS, their success rate is not always guaranteed. The severity of IFIS varies greatly among patients, and the condition can be unpredictable in many cases [9].

An ophthalmologist and a urologist must monitor IFIS. The disease involves the eyes and prostate, prevalent conditions in old age [2].

While the risks and complications are high for some, it is important to remember that IFIS is rare. As of 2009, only 2% of cataract surgeries were influenced by this condition [10].

Precautions and education about IFIS are important for the patient and health care providers. The ophthalmologist and/or urologist should instruct the patients about the risks and management of IFIS before undergoing cataract surgery. Patients predisposed to the appearance of cataracts should be evaluated and referred to an ophthalmologist to determine the operative measure before starting the treatment for BPH and competently assess this medicine’s administration [2,7].

Tamsulosin administration is the most common cause of IFIS [11,12]. However, not all patients who received tamsulosin develop IFIS and cases without tamsulosin treatment have been reported.

Among other possible side effects of alpha-blockers, we mention: vertigo, headache, palpitations, arterial hypotension, allergies, abnormal ejaculation, priapism, dry mouth, blurred vision, and nosebleeds [9]. Patients predisposed to cataracts should be evaluated and referred to an ophthalmologist to schedule surgery before starting the treatment for BPH and competently assess this medicine’s administration [7,2].

## Conclusions

Collaboration between urologists and ophthalmologists is required for patients prone to the appearance of cataracts since both conditions are found in elderly patients.

In our case report, the frequency of surgical complications related to the administration of tamsulosin was 3%. No major complications occurred.

Patients predisposed to the appearance of cataracts should be evaluated professionally regarding this medicine’s administration.

A floppy iris undulates and moves excessively when fluids are injected into the eye during cataract surgery, leading to complications and great stress on the surgeon. But what happens to other tissues that cannot be seen like the iris?

## References

[1] Chang DF, Campbell JR (2005). Intraoperative floppy iris syndrome associated with tamsulosin. J Cataract Refract Surg.

[2] Zaman F, Bach C, Junaid I, Papatsoris AG, Pati J, Masood J, Buchholz N (2012). The Floppy Iris Syndrome–What Urologists and Ophthalmologists Need to Know. Current Urology.

[3] Chatziralli IP, Peponis V, Parikakis E, Maniatea A, Patsea E, Mitropoulos P (2016). Risk factors for intraoperative floppy iris syndrome: a prospective study. Eye.

[4] Chang DF, Osher RH, Wang L, Koch DD (2007). Prospective multicenter evaluation of cataract surgery in patients taking tamsulosin. Ophthalmology.

[5] Christou CD, Tsinopoulos I, Ziakas N, Tzamalis A (2020). Intraoperative Floppy Iris Syndrome: Updated Perspectives. Review Clin Ophthalmol.

[6] Christou CD, Tsinopoulos I, Ziakas N (2020). Intraoperative Floppy Iris Syndrome: Updated Perspectives. Clin Ophthalmol.

[7] Keklikci U, Isen K, Unlu K (2009). Incidence, clinical findings, and management of intraoperative floppy iris syndrome associated with tamsulosin. Acta Ophthalmol.

[8] Xue Yang, Zhaochuan Liu, Zhigang Fan, Andrzej Grzybowski, Ningli Wang A narrative review of intraoperative floppy iris syndrome: an update 2020.

[9] Bell CM, Hatch WV, Fischer HD, Cernat G, Paterson JM, Gruneir A, Gill SS, Bronskill Anderson GM, Rochon PA (2009). Association between tamsulosin and serious ophthalmic adverse events in older men following cataract surgery. JAMA.

[10] Takmaz T, Can I (2007). Clinical features, complications, and incidence of intraoperative floppy iris syndrome in patients taking tamsulosin. Eur J Ophthalmol.

[11] Tiwari A (2006). Tamsulosin and floppy iris syndrome in benign prostatic hyperplasia patients. Expert Opin Investig Drugs.

[12] Shah N, Tendulkar M, Brown R (2009). Should we anticipate intra-operative floppy iris syndrome (IFIS) even with very short history of tamsulosin?. Eye.

